# Diagnosis and surgical strategy for sacral meningeal cysts with check-valve mechanism: technical note

**DOI:** 10.1007/s00701-012-1550-7

**Published:** 2012-11-17

**Authors:** Shunji Asamoto, Yasuyuki Fukui, Makoto Nishiyama, Masayuki Ishikawa, Nobuyuki Fujita, Satoshi Nakamura, Jun Muto, Yuta Shiono, Hiroshi Doi, Motoo Kubota, Kazuhiko Ishii

**Affiliations:** 1 Spine and Spinal Cord Center, International University of Health and Welfare Mita Hospital, 1-4-3 Mita, Minatoku, Tokyo, 108-8329 Japan; 2 Department of Neurosurgery, Tokyo Metropolitan Ebara Hospital, Tokyo, Japan; 3 Department of Neurosurgery, Kameda General Hospital, Kamogawa, Japan; 4 Department of Neurosurgery, Faculty of Medicine, The University of Tokyo, Tokyo, Japan

**Keywords:** Sacral meningeal cyst, Check-valve mechanism, Surgical strategy

## Abstract

**Objective:**

There is agreement that symptomatic sacral meningeal cysts with a check-valve mechanism and/or large cysts representing space-occupying lesions should be treated surgically. This study investigated factors indicating a need for surgical intervention and surgical techniques for sacral meningeal cysts with a check-valve mechanism.

**Methods:**

In ten patients presenting with sciatica and neurological deficits, myelography, computed tomography (CT) myelography, and magnetic resonance imaging (MR imaging) detected sacral meningeal cysts with a check-valve mechanism. One patient had two primary cysts. Ten cysts were type 2 and one cyst was type 1. Nine of the ten patients had not undergone previous surgery, while the remaining case involved recurrent cyst. For the seven patients with normal (i.e., not huge or recurrent) type 2 cysts and no previous surgery (eight cysts), suture after collapse of the cyst wall was performed. For the recurrent type 2 cyst, duraplasty and suture with collapse of the cyst wall were performed to eliminate the check-valve mechanism. For the remaining type 2 cyst, a primary root was sacrificed because of the huge size of the cyst. For the type 1 cyst, the neck of the cyst was ligated.

**Results:**

In all cases, chief complaints disappeared immediately postoperatively and no deterioration of clinical symptoms has been seen after a mean follow-up of 27 months.

**Conclusions:**

The presence or absence of a check-valve mechanism is very important in determining the need for surgical intervention for sacral meningeal cysts.

## Introduction

A sacral meningeal cyst is often found incidentally, and no specific surgical method has been established for most cases [9, 14]. With large and/or sacral meningeal cysts with a check-valve mechanism, surgery must occasionally be performed [5]. Here we report ten operative cases of sacral meningeal cyst with a check-valve mechanism.

## Materials and methods

**Fig. 1:**
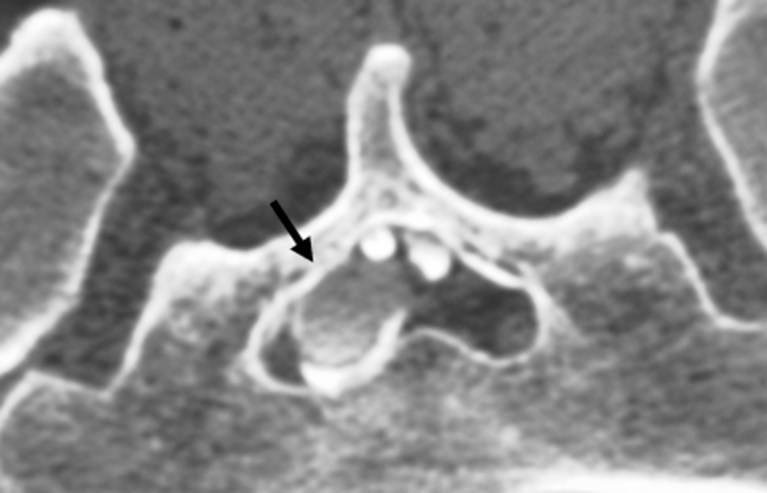
CT myelography in case 8 (prone position, caudal view) reveals the primary cyst (*arrow*). Contrast medium shows only weak filling in the primary cyst

**Fig. 2:**
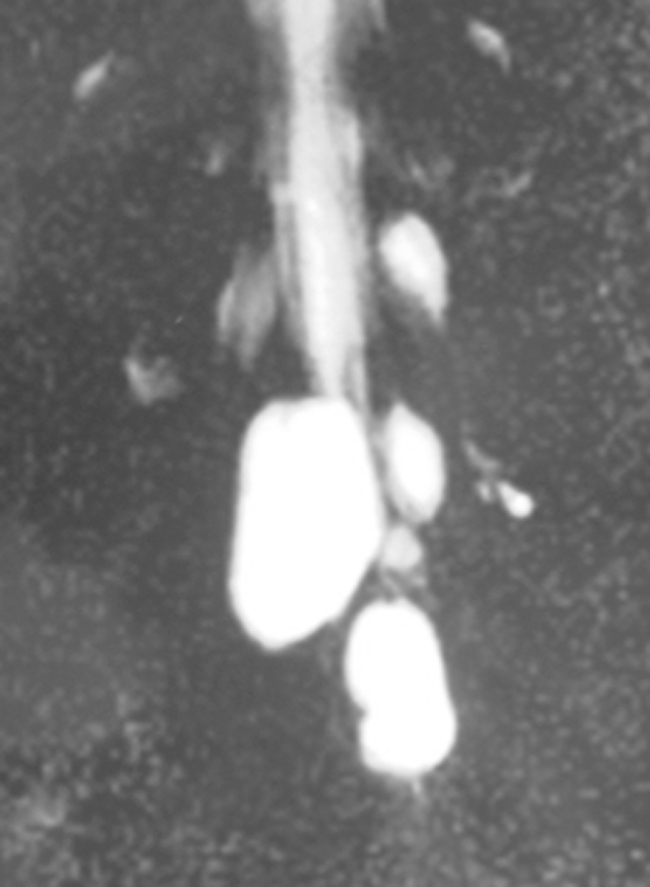
MR myelography in case 8 (MRM) showing multiple sacral meningeal cysts. However, the primary cyst cannot be identified from MRM

Participants in this study comprised six men and four women (mean age, 42.1 years; range, 18–56 years). Myelography, computed tomography (CT) myelography, and magnetic resonance imaging (MRI) were performed preoperatively in all the cases. CT myelography is essential to clarify the bone structure and the relationships with the cyst and the bone. Multiple cysts were observed in all cases, and myelography and CT myelography were required to identify the primary cyst. In nine cases, only one primary cyst was present (Figs. 1, 2, and 4). The remaining case showed two primary cysts (case 9, Table 1). All primary cysts were classified using Nabors’ classification [8], which is based on the anatomical location of the cyst and the relationships to surrounding nerve roots, as follows: type 1, extradural cyst without nerve root fiber; type 2, extradural cyst with nerve root fiber; and type 3, intradural cyst. For extradural cysts without the presence of a nerve root fiber (type 1), the cyst wall was sharply incised and ligated by suturing to remove the check-valve under the microscope and prevent injury to the nerve with careful observation. For extradural cysts with nerve root fiber (type 2), the cyst wall is opened and the nerve root identified. Where the root passes through the foramen, the check-valve system can be confirmed using the Valsalva maneuver. Part of the cyst wall is then removed and sutured tightly. Especially in cases of huge cysts, care should be taken to avoid leaving space when suturing the wall. The use of continuous sutures with non-absorbable thread is prefered.

**Table 1:** Summary of patients. Case 7 involved recurrent type 2 cyst. Case 9 showed two primary type 2 cysts. Case 10 is a huge type 2 cyst

Case	Age (years)	Sex	Primary	Type	Main symptoms	Check-valve	Operations	Follow-up (years)
1	40	M	Rt. S2	1	Rt. sciatica, leg hypesthesia	+	Ligation of neck of the cyst	16
2	52	M	Rt. S2	2	Rt. sciatica, leg hypesthesia	+	Suture after collapse of cyst wall	15
3	18	M	Rt. S2	2	Rt. sciatica, leg hypesthesia	+	Suture after collapse of cyst wall	15
4	44	M	Lt. S3	2	Lt. sciatica, leg hypesthesia, coccydynia	+	Suture after collapse of cyst wall	12
5	53	F	Rt. S3	2	Rt. sciatica, leg hypesthesia, coccydynia	+	Suture after collapse of cyst wall	12
6	42	F	Lt. S2	2	Lt. sciatica, leg hypesthesia, coccydynia	+	Suture after collapse of cyst wall	10
7	56	M	Rt. S2	2	Rt. sciatica, leg hypesthesia, sphincter-bowel dysfunction, weakness of right gastrocnemius muscle	+	Suture after collapse of cyst wall, elimination of check-valve with duraplasty	7
8	33	M	Lt. S2	2	Lt. sciatica, leg hypesthesia	+	Suture after collapse of cyst wall	7
9	54	F	Lt. S2	2	bilateral sciatica, leg hypesthesia, sphincter-bowel dysfunction, weakness of right FHL	+	Suture after collapse of cyst wall	7
			Rt. S2	2		+	Suture after collapse of cyst wall	6
10	29	F	Lt. S3	2	Lt. sciatica, leg hypesthesia	+	Sacrifice of Lt. S3 root	6

Type: Nabors’ classification (Nabors MW et al. 1988). *Rt.* right; *Lt.* left; *FHL* flexor hallucis longus

**Fig. 3:**
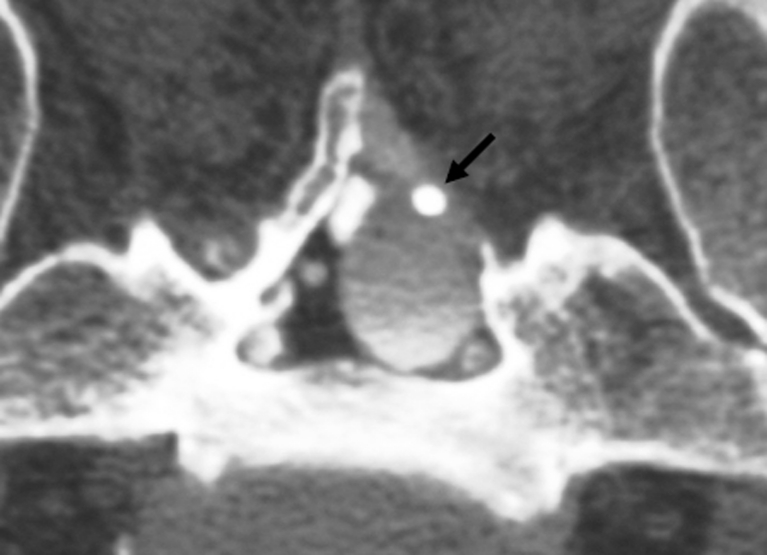
Recurrent cyst in case 7. A malfunctioning shunt tube is detected in the cyst (*arrow*). Contrast medium in the cyst shows low density

**Fig. 4:**
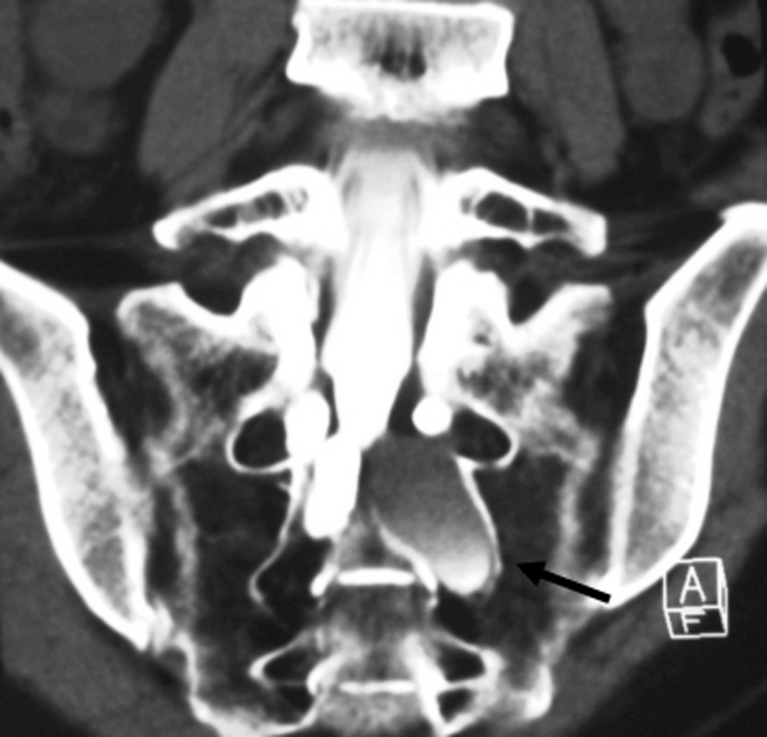
Coronal CT myelography in case 6, allowing easy identification of primary cyst (*arrow*)

Nine cases (ten cysts) were type 2 and one case was a type 1 sacral meningeal cyst. Of the ten patients, nine had not undergone any previous surgery for cysts. The remaining patient showed recurrent cyst. In this recurrent case, cyst-subarachnoid shunt had been performed 8 years earlier, but the shunt system had started to malfunction (Fig. 3). All ten patients (11 cysts) underwent surgery. All patients had presented with sciatica, bowel sphincter dysfunction, or motor weakness of the lower extremities. In all except the type 1 cyst, the primary cyst was not symptomatic, because the primary cyst did not compress other roots in the spinal canal (Fig. 4).

### Operations

**Fig. 5:**
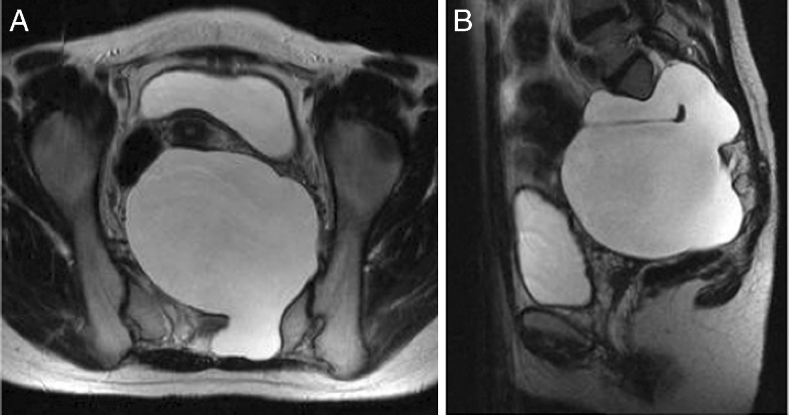
**a**, **b** T2-weighted MRI in case 10 revealing a huge presacral mass (11 × 10 × 9 cm). The huge sacral meningeal huge enters the presacral space through the left S2 and S3 foramina

**Fig. 6:**
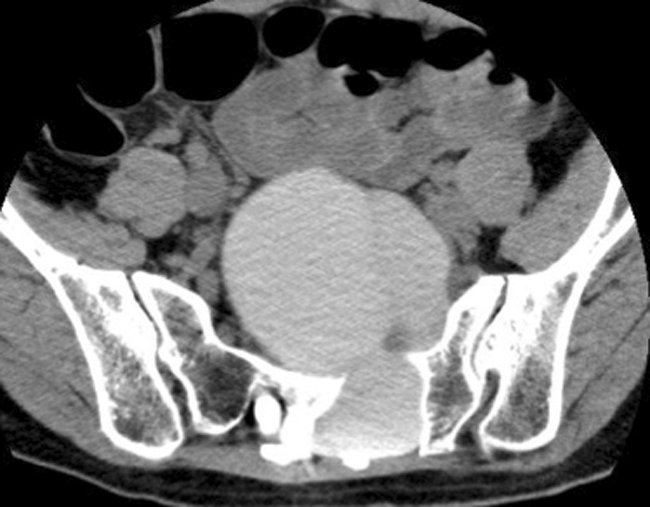
CT myelography in case 10 showing the huge cyst (supine position, caudal view). The left S2 root is compressed and the S3 root is continuous with the cyst interior

For the seven patients with normal (i.e., not recurrent or huge) type 2 cyst and no previous surgery (eight cysts), suturing the cyst wall after collapse of the cyst wall was performed. For the recurrent case, duraplasty and suture after collapse of the cyst wall were performed to eliminate the check-valve mechanism of the cyst. For the remaining type 2 cyst without previous surgery, the primary root was sacrificed under meticulous monitoring because of the huge size of the cyst (Figs. 5 and 6). For the type 1 cyst, ligation of the neck of the cyst was performed (Table 1).

## Results

Perioperative complications were not observed in any except the recurrent case. In that case, subcutaneous leakage of cerebrospinal fluid (CSF) occurred 2 weeks after surgical intervention. After spinal drainage was performed for 4 weeks, CSF leakage disappeared completely. In all cases, the chief complaints disappeared immediately postoperatively. All cases were followed-up after the operation (range of follow-up, 9 months to 6 years), with no further recurrences identified in any cases. In the seven cases (eight cysts) of normal type 2 cyst with no previous surgeries, the check-valve mechanism was eliminated naturally (automatic elimination) after collapse of the cyst wall and suturing. Elimination of the check-valve mechanism could be confirmed when CSF began to flow naturally through the cyst.

## Discussion

Due to advances in MRI, sacral meningeal cysts are increasingly being incidentally [10, 13, 15]. Most of the time, sacral meningeal cysts do not have a typical presentation [11], and operable cases are rare. In most cases presenting with multiple cysts, surgery is indicated for only one of the cysts [4]. The most important task in the case of multiple cysts is identification of the primary cyst by myelography and CT myelography [12, 15]. In the primary cyst, contrast medium does not flow into the cyst immediately after injection into the subarachnoid space. CT myelography reveals only a weak filling of contrast medium into the primary cyst, and also confirms differences in the density of contrast medium among cysts in the case of multiple cysts or from the degree of contrast filling in the subarachnoid space. The primary cyst is the one with the check-valve mechanism [6]. Because pressure within the cyst is greater than that in the subarachnoid space, contrast medium does not flow easily into the cyst. The check-valve mechanism is responsible for the one-way flow of CSF from the caudal sac to the cyst, which is a good indication for surgery [6]. Primary and secondary cysts can thus be confirmed on the basis of differences in the density of contrast medium. In our experience, most cysts showing a check-valve mechanism are type 2 cysts.

**Fig. 7:**
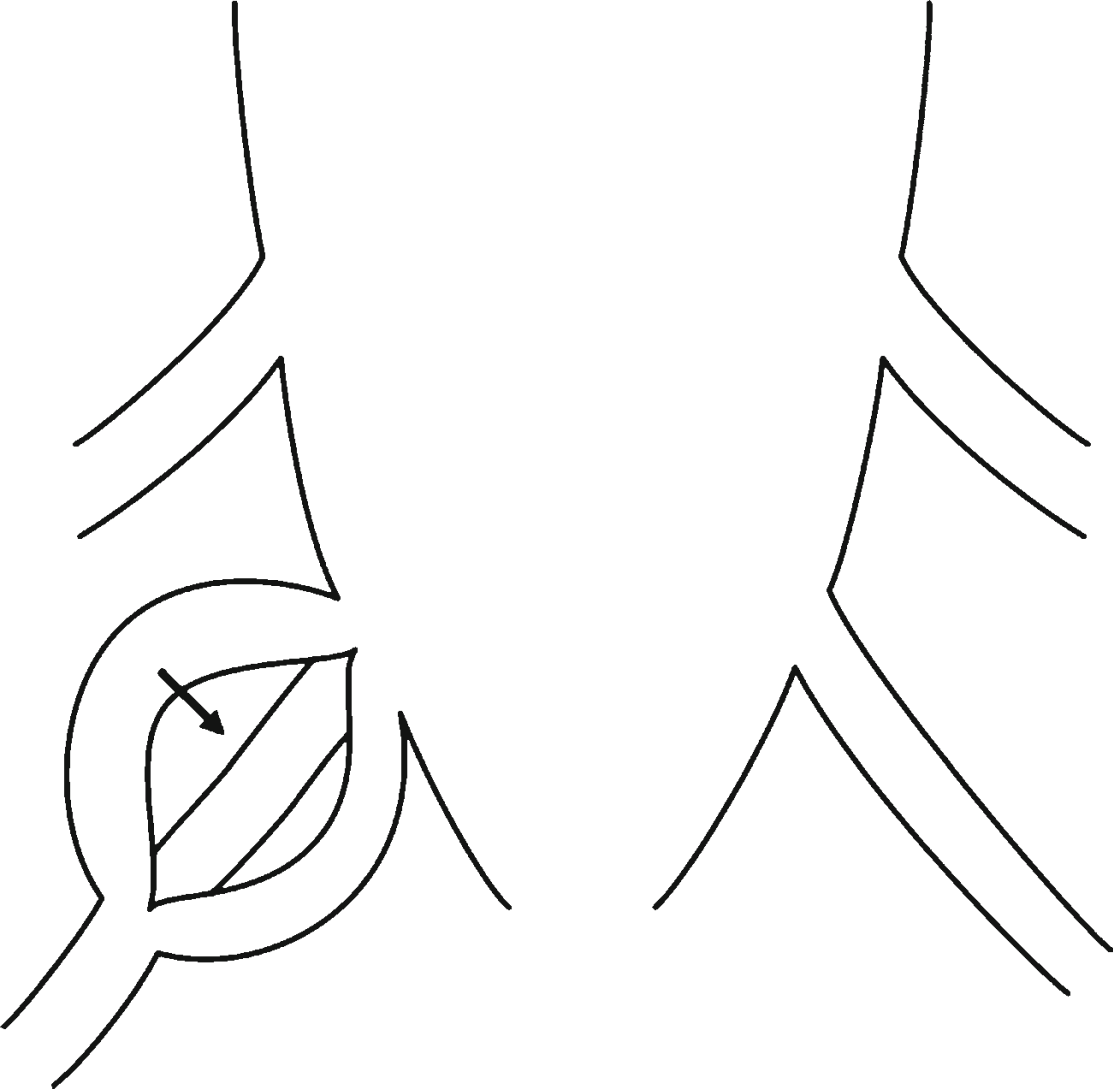
Schematic image of a type 2 cyst. After opening the cyst, the root is identified (*arrow*)

**Fig. 8:**
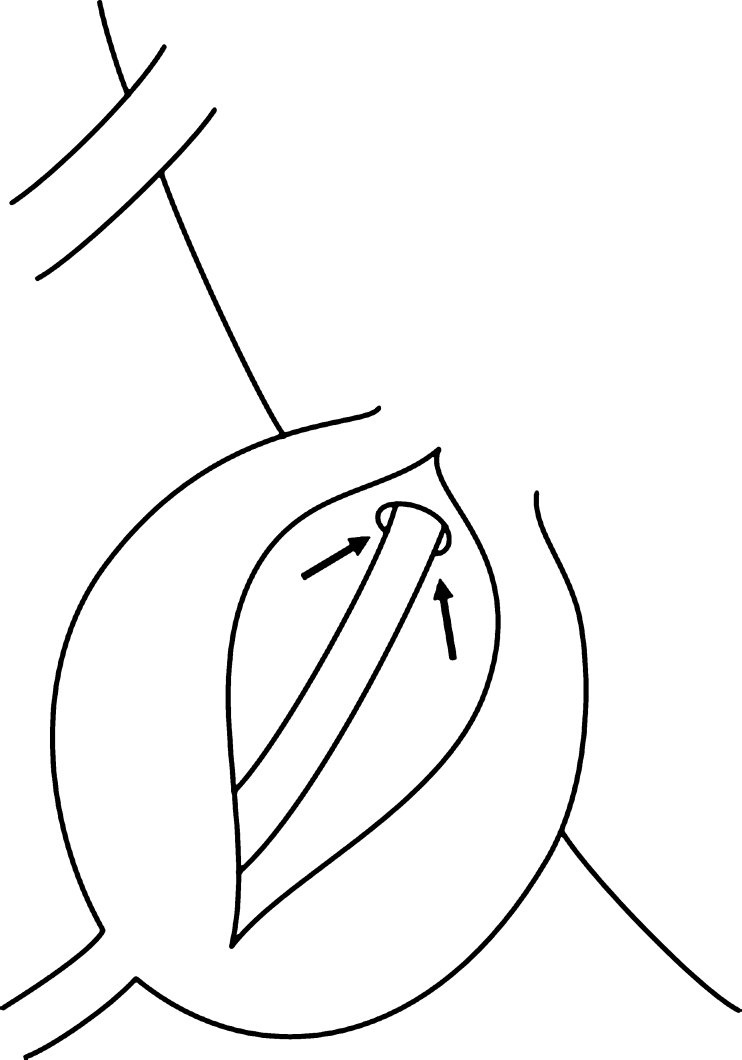
Schematic image of the “check-valve mechanism” (*arrow*). During the operation, the “check-valve mechanism” should be detected using the Valsalva method

**Fig. 9:**
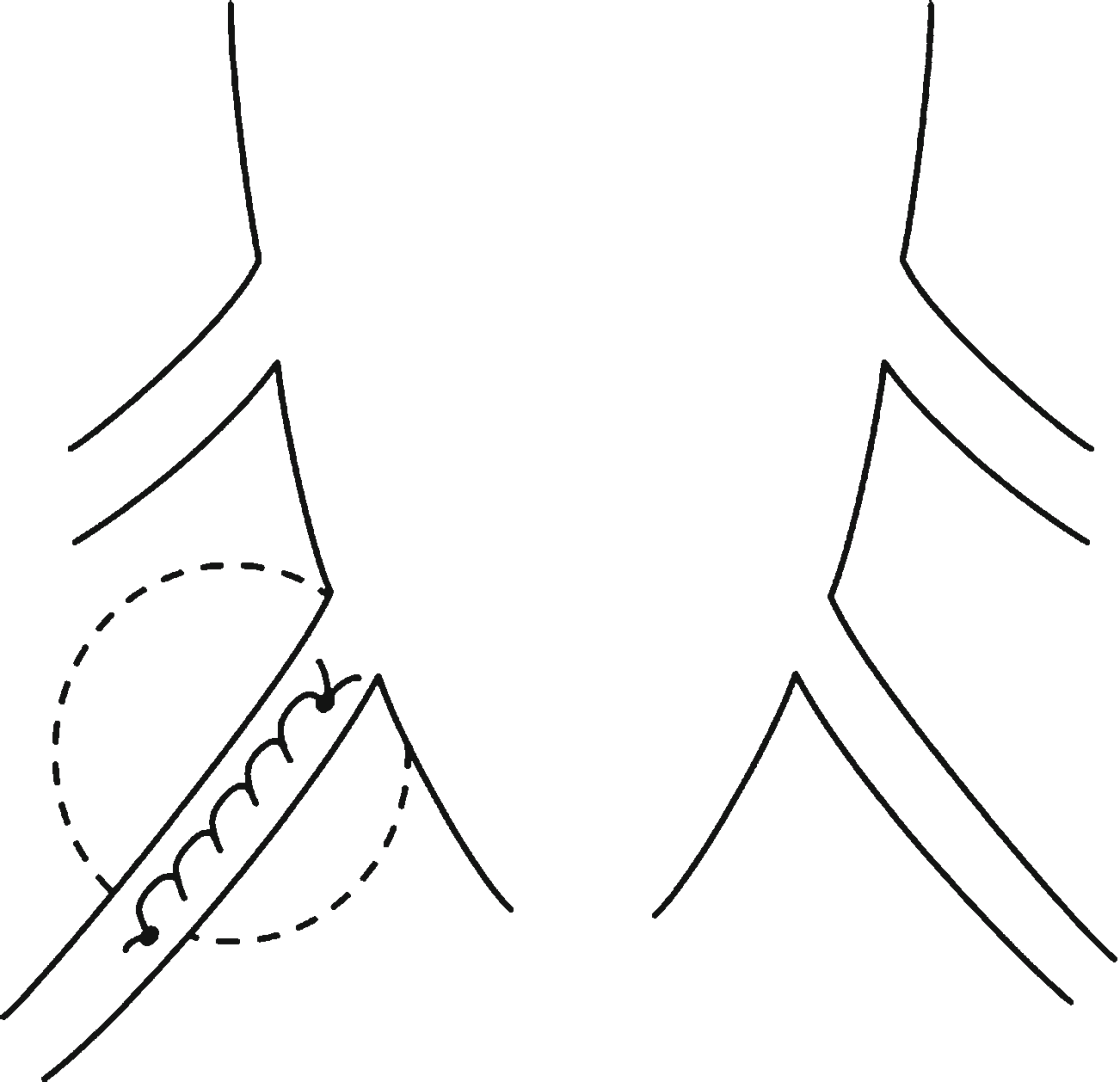
Schematic image of the suture with collapse of the cyst wall, as in “nerve root plasty”

**Fig. 10:**
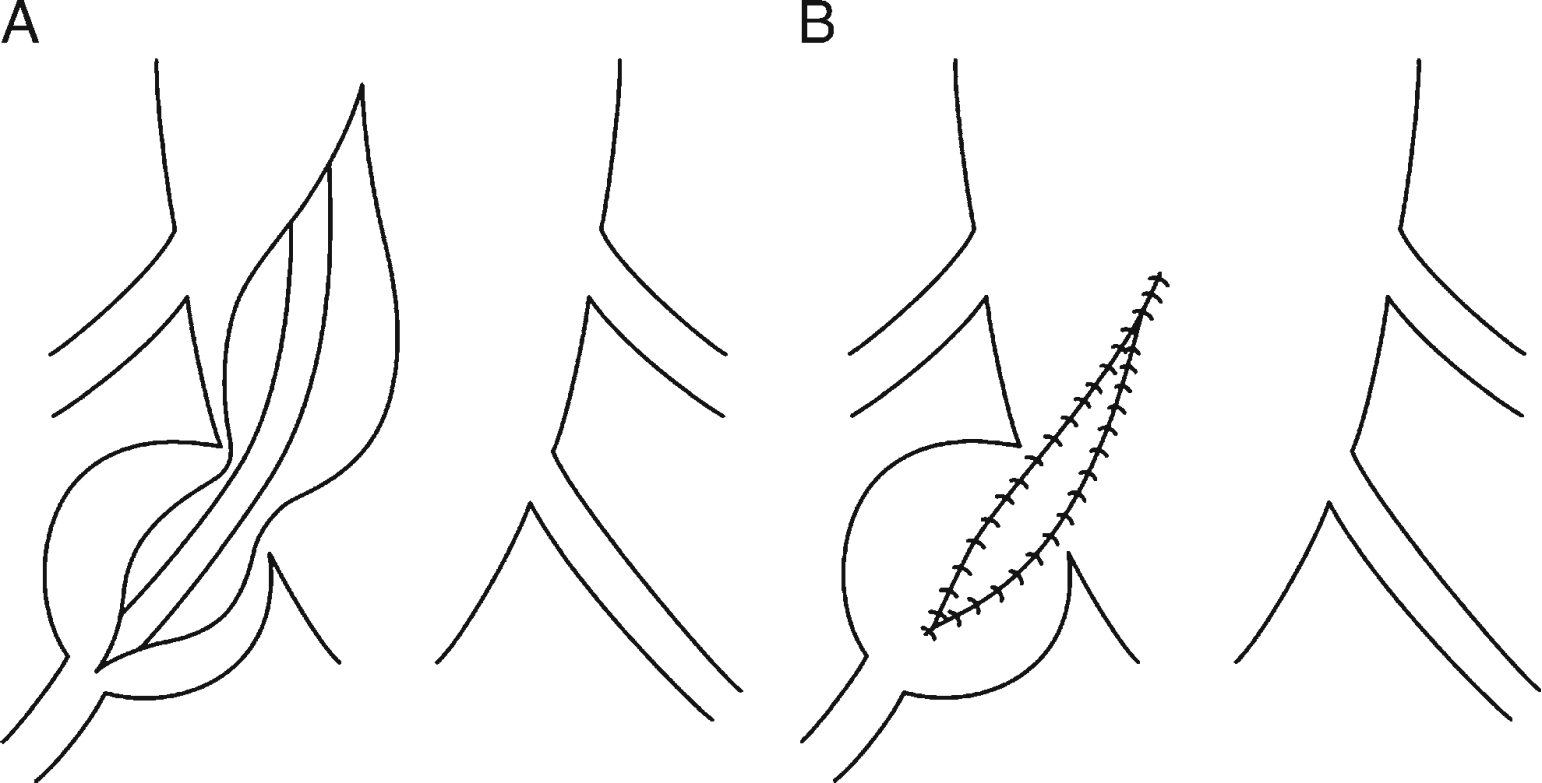
Schematic of elimination of the “check-valve mechanism” (**a**) and elimination with GORE-TEX (**b**)

Previous reports have mentioned the use of various operative methods for cysts with check-valve mechanisms [1–4, 6, 7, 12]. The operative method used in this case involves performing a cyst-subarachnoid shunt, suturing after collapse of the cyst wall, and plugging of a piece of muscle tissue in the region from where the root penetrates the cyst. However, the shunt might become occluded over time, and the plugging with muscle may lead to inflammatory changes at the root, resulting in unfavorable outcomes. The main aim of performing this operation is to eliminate the check-valve mechanism, which develops in the area where the root arises from the caudal sac towards the cyst. Our experience shows that it is possible to confirm elimination of the check-valve mechanism intraoperatively. The cyst should be opened and the origin of the nerve root confirmed before collapsing and suturing the cyst wall (Fig. 7). The position of the check-valve mechanism should then be confirmed. The Valsalva method is helpful to identify this point (Fig. 8). Many variations in CSF inflow pattern into the cyst can be seen due to the check-valve mechanism. A jet flow through the leakage point can be observed in some cases, while other cases may show no CSF flow during the Valsalva maneuver. The check-valve mechanism was eliminated naturally when sutured the collapsed cyst wall in all cases except those involving reoperation or huge cyst. Because the cyst was treated by suturing after collapsing the cyst wall from which the root arises, the check-valve mechanism was thought to be eliminated naturally. Importantly, suturing after collapse of the cyst wall should be performed “like a nerve root plasty” (Fig. 9). Furthermore, it is necessary to evaluate whether the root of a large cyst can be sacrificed under meticulous monitoring. In addition, suture after collapse of the cyst wall is difficult in re-operated cases, and the method of eliminating the check-valve mechanism directly using artificial GORE-TEX appears to warrant investigation (Fig. 10).

## Conclusions

Generally, the check-valve mechanism is eliminated naturally after suturing and collapse of the cyst wall. However, duraplasty must be performed in recurrent cases, when the check-valve mechanism has not been eliminated and surgical removal is required to establish communication with the caudal sac. The presence or absence of a check-valve mechanism is very important in determining whether surgical intervention for the sacral meningeal cyst is necessary.
